# Supplementary material for: Combined Inhibitory Effect of Canada Goldenrod Invasion and Soil Microplastics on Rice Growth
Source: Int J Environ Res Public Health. 2022 Sep 21;19(19):11947. doi: 10.3390/ijerph191911947 (PMC9565921; doi:10.3390/ijerph191911947)
Supplement: Supplementary file 1 [file ijerph-19-11947-s001.zip › ijerph-1927894-supplementary.pdf]

Table S1. RMANOVA results of rice belowground, aboveground, and total biomass under different treatments during the study period.

Treatment	Belowground biomass		Aboveground biomass		Total biomass	
	F	*P*	F	*P*	F	*P*
SI	442.60	<0.001	89.44	< 0.001	448.76	< 0.001
MPs	2060.97	<0.001	243.25	< 0.001	1359.97	< 0.001
SI × MPs	15.56	<0.001	0.86	0.363	11.65	<0.001
DAT	4378.80	<0.001	789.26	< 0.001	3946.93	< 0.001
DAT × SI	15.49	< 0.001	37.83	< 0.001	183.02	< 0.001
DAT × MPs	67.83	< 0.001	63.97	< 0.001	305.24	< 0.001
DAT × SI × MPs	35.45	< 0.001	0.77	0.389	10.66	0.003

DAT = days after transplanting; CK = control treatment; SI = *Solidago canadensis* L. invasion treatment; MPs = soil microplastics residual treatment; SI × MPs = combination of *S. canadensis* invasion treatment and soil microplastics residual treatment.

Table S2. The RMANOVA results for rice phenotypic indices under different treatments during the study period.

Treatment	Stem height		Total height		Diameter		No., of leaves		No., of tillers		No., of nodes	
	F	*P*	F	*P*	F	*P*	F	*P*	F	*P*	F	*P*
SI	27.80	< 0.001	14.38	< 0.001	23.41	< 0.001	0.04	0.836	23.21	< 0.001	4.59	0.043
MPs	59.71	< 0.001	39.97	< 0.001	50.92	< 0.001	4.80	0.004	32.89	< 0.001	9.11	0.006
SI × MPs	0.02	0.899	1.38	0.251	5.17	0.003	0.88	0.357	2.58	0.121	0.77	0.389
DAT	67.36	< 0.001	3.16	0.088	57.63	< 0.001	20.44	< 0.001	13.93	0.001	3.62	0.069
DAT × SI	3.42	0.076	0.01	0.910	1.59	0.219	0.35	0.561	0.03	0.860	6.19	0.020
DAT × MPs	2.89	0.102	0.23	0.632	7.16	0.013	2.17	0.153	7.11	0.014	6.94	0.015
DAT × SI × MPs	0.04	0.847	0.59	0.449	0.02	0.882	0.24	0.6276	0.03	0.860	2.14	0.156

DAT = days after transplanting; CK = control treatment; SI = *Solidago canadensis* L. invasion treatment; MPs = soil microplastics residual treatment; SI × MPs = combination of *S. canadensis* invasion treatment and soil microplastics residual treatment.

Table S3. The RMANOVA results for rice photosynthetic parameters under different treatments during the study period.

Treatment	Pn		Tr		Gs		PAR		Ci		WUE		SPAD		Leaf N	
	F	*P*	F	*P*	F	*P*	F	*P*	F	*P*	F	*P*	F	*P*	F	*P*
SI	164.63	<0.001	287.28	<0.001	410.79	<0.001	159.45	<0.001	339.15	<0.001	105.70	<0.001	248.53	<0.001	23.42	<0.001
MPs	936.52	<0.001	899.26	<0.001	1864.65	<0.001	623.00	<0.001	1332.91	<0.001	212.61	<0.001	716.58	<0.001	77.66	<0.001
SI × MPs	4.35	0.048	26.38	<0.001	5.39	0.029	2.24	0.148	0.01	0.929	7.17	0.013	19.62	<0.001	0.69	0.416
DAT	51.71	<0.001	276.30	<0.001	35.22	<0.001	156.05	<0.001	15002.10	<0.001	12.02	0.002	0.04	0.839	0.88	0.358
DAT × SI	1.59	0.22	7.36	0.012	0.26	0.611	18.19	<0.001	298.71	<0.001	2.85	0.104	1.66	0.210	11.01	0.003
DAT × MPs	0.73	0.403	0.43	0.520	1.13	0.298	14.00	0.001	1182.76	<0.001	9.04	0.006	16.57	<0.001	32.91	<0.001
DAT × SI × MPs	4.49	0.045	6.45	0.018	45.55	<0.001	5.08	0.034	0	0.965	2.95	0.099	0.04	0.839	1.09	0.308

DAT = days after transplanting; CK = control treatment; SI = *Solidago canadensis* L. invasion treatment; MPs = soil microplastics residual treatment; SI × MPs = combination of *S. canadensis* invasion treatment and soil microplastics residual treatment; Pn = net photosynthetic rate; Tr = transpiration rate; Gs = stomatal conductance; PAR = photosynthetically active radiation; Ci = intracellular CO_2 concentration; WUE = water-use efficiency; Leaf N = leaf nitrogen.

Table S4. The RMANOVA results for antioxidants enzymes activities and reactive oxygen species in the belowground and aboveground parts of the rice under different treatments during the study period.

Parts	Treatment	APX		CAT		POD		SOD		ROS	
		F	*P*	F	*P*	F	*P*	F	*P*	F	*P*
Belowground	SI	16.95	<0.001	16.10	<0.001	0.60	0.450	60.28	<0.001	10.92	0.003
	MPs	58.86	<0.001	54.76	<0.001	97.86	<0.001	221.89	<0.001	26.85	<0.001
	SI × MPs	0.52	0.477	1.94	0.180	45.90	<0.001	1.60	0.220	2.30	0.140
	DAT	44.09	<0.001	1.10	0.300	282.22	<0.001	239.36	<0.001	430.14	<0.001
	DAT × SI	0.11	0.747	0.01	0.908	0.83	0.373	27.85	<0.001	18.83	<0.001
	DAT × MPs	7.21	0.013	0.08	0.783	35.00	<0.001	83.27	<0.001	48.78	<0.001
	DAT × SI × MPs	0	0.969	0.09	0.764	0.14	0.716	0.67	0.420	0.04	0.841
Aboveground	SI	8.01	0.009	8.48	0.008	1.74	0.200	92.08	<0.001	32.37	<0.001
	MPs	0.52	0.480	33.63	<0.001	6.25	0.020	233.57	<0.001	121.32	<0.001
	SI × MPs	55.94	<0.001	0.88	0.360	3.23	0.080	20.91	<0.001	4.79	0.039
	DAT	41.42	0.001	73.75	<0.001	15.94	<0.001	248.27	<0.001	5.40	0.029
	DAT × SI	0.81	0.380	1.38	0.252	22.98	<0.001	11.53	0.002	0	0.990
	DAT × MPs	0.72	0.400	7.32	0.012	75.18	<0.001	38.78	<0.001	1.06	0.310
	DAT × SI × MPs	12.69	0.002	0.02	0.902	0.02	0.890	8.75	0.007	2.51	0.120

DAT = days after transplanting; CK = control treatment; SI = *Solidago canadensis* L. invasion treatment; MPs = soil microplastics residual treatment; SI × MPs = combination of *S. canadensis* invasion treatment and soil microplastics residual treatment; APX = ascorbate peroxidase; CAT = catalase; POD = peroxidase; SOD = superoxide dismutase; ROS = reactive oxygen species.
